# Milk protects against sarcopenic obesity due to increase in the genus *Akkermansia* in faeces of *db/db* mice

**DOI:** 10.1002/jcsm.13245

**Published:** 2023-05-02

**Authors:** Takuro Okamura, Masahide Hamaguchi, Hanako Nakajima, Nobuko Kitagawa, Saori Majima, Takafumi Senmaru, Hiroshi Okada, Emi Ushigome, Naoko Nakanishi, Ryoichi Sasano, Michiaki Fukui

**Affiliations:** ^1^ Department of Endocrinology and Metabolism Kyoto Prefectural University of Medicine, Graduate School of Medical Science Kyoto Japan; ^2^ AiSTI SCIENCE CO., Ltd. Wakayama Japan

**Keywords:** *Akkermansia muciniphila*, cow milk, faecal microbiota transplantation, milk, sarcopenic obesity

## Abstract

**Background:**

Sarcopenic obesity, a combination of sarcopenia and obesity, is a pathological feature of type 2 diabetes. Several human studies have shown that milk is useful in the prevention of sarcopenia. This study was aimed at clarifying the effect of milk on the prevention of sarcopenic obesity in *db/db* mice.

**Methods:**

A randomized and investigator‐blinded study was conducted using male *db/db* mice. Eight‐week‐old *db/db* mice were housed for 8 weeks and fed milk (100 μL/day) using a sonde. The faecal microbiota transplantation (FMT) group received antibiotics for 2 weeks, starting at 6 weeks of age, followed by FMT twice a week until 16 weeks of age.

**Results:**

Milk administration to *db/db* mice increased grip strength (Milk−: 164.2 ± 4.7 g, Milk+: 230.2 ± 56.0 g, *P* = 0.017), muscle mass (soleus muscle, Milk−: 164.2 ± 4.7 mg, Milk+: 230.2 ± 56.0 mg, *P* < 0.001; plantaris muscle, Milk−: 13.3 ± 1.2 mg, Milk+: 16.0 ± 1.7 mg, *P* < 0.001) and decreased visceral fat mass (Milk−: 2.39 ± 0.08 g, Milk+: 1.98 ± 0.04 mg, *P* < 0.001), resulting in a significant increase in physical activity (light: *P* = 0.013, dark: *P* = 0.034). FMT from mice fed milk not only improved sarcopenic obesity but also significantly improved glucose intolerance. Microarray analysis of gene expression in the small intestine revealed that the expression of amino acid absorption transporter genes, namely, *SIc7a5* (*P* = 0.010), *SIc7a1* (*P* = 0.015), *Ppp1r15a* (*P* = 0.041) and *SIc7a11* (*P* = 0.029), was elevated in mice fed milk. In 16S rRNA sequencing of gut microbiota, the genus *Akkermansia* was increased in both the mice fed milk and the FMT group from the mice fed milk.

**Conclusions:**

The findings of this study suggest that besides increasing the intake of nutrients, such as amino acids, milk consumption also changes the intestinal environment, which might contribute to the mechanism of milk‐induced improvement of sarcopenic obesity.

## Introduction

The incidence of type 2 diabetes mellitus is increasing worldwide.^1^ Overeating^2^ and inactivity^3^ are the main causes of type 2 diabetes, and the associated increase in visceral fat and decrease in muscle mass or sarcopenia^4^ and obesity are the pathological bases for this disease. We previously reported findings of various human and animal studies on sarcopenic obesity and type 2 diabetes and showed that *db/db* mice with defects in the leptin receptor, characterized by overeating and inactivity, exhibit sarcopenic obesity.^5^ Thus, prevention of sarcopenic obesity is important to prevent the onset and exacerbation of type 2 diabetes.

Nutrition and exercise interventions are generally recommended as preventive measures for sarcopenia.^6^ For nutritional intervention, milk and dairy products may be beneficial, and their consumption is effective in maintaining and improving muscle mass and muscle function.^7^ Whole cow milk contains high‐quality protein (20% whey and 80% casein), minerals (calcium, phosphorus, magnesium, iodine etc.), vitamins (fat‐soluble vitamins A and E, water‐soluble vitamin B etc.), carbohydrates (lactose and oligosaccharides) and fat (70% saturated fatty acids, 30% monounsaturated fatty acids and polyunsaturated fatty acids).^8^ Exercise‐induced injury, muscle soreness and post‐exercise performance loss were reduced in young men and women who consumed whole milk instead of an isocaloric carbohydrate drink after exercise.^9^ However, in a narrative review of three observational and eight intervention studies that used milk, with or without exercise, as an intervention to promote muscle function in older adults, the correlation of milk with sarcopenia was negative in intervention studies, and the studies in which a combination of milk and exercise was used were inconclusive.^10^ Although milk contains muscle‐protective nutrients, the current evidence does not indicate a beneficial effect of milk on muscle health in elderly patients. There are differences in the background of target patients and in the duration of administration, which might be the cause of discordance in results.

There have been various reports on the association between sarcopenia and the gut microbiota.^11^ For example, supplementation with *Lactobacillus acidophilus* and *Bifidobacterium bifidum* markedly improved muscle mass, strength and endurance in aged mice.^12^ The gut–muscle axis may provide novel targets for age‐related muscle wasting and dysfunction. Several animal studies have reported metabolic alterations induced by cow milk administration that involves not only nutrient supplementation but also various modifications of the gut microbiota.^13^ However, there have been no studies to date that have shown the effects of faecal microbiota transplantation (FMT) altered by milk on muscle.

In this study, we aimed to clarify the antisarcopenic obesity effect of milk intake using *db/db* mice, a mouse model of sarcopenic obesity, after FMT from mice fed milk.

## Methods

### Mice

All experimental procedures were approved by the Committee for Animal Research, Kyoto Prefectural University of Medicine, Japan (Approval Number M2022‐84). A randomized and investigator‐blinded study was conducted using male *db/db* mice.

#### Stage 1

**Figure 1:**
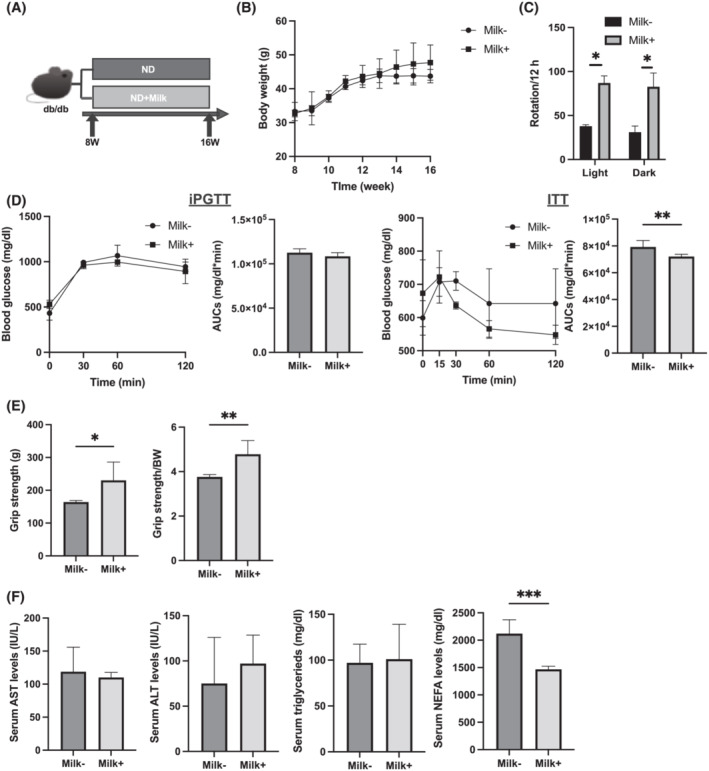
Administration of milk increased grip strength. (A) Administration of milk started at 8 weeks of age, and mice were sacrificed at 16 weeks of age. (B) Changes in the body weight (*n* = 6). (C) The numbers of rotation in the light and dark phases using the running wheel (*n* = 6). (D) Results of intraperitoneal glucose tolerance testing (iPGTT; 2 g/kg of body weight) for 15‐week‐old mice and the area under the curve (AUC) analysis (*n* = 6). Results of insulin tolerance testing (ITT; 0.5 U/kg of body weight) for 15‐week‐old mice and the AUC analysis (*n* = 6). (E) Absolute and relative grip strength (*n* = 6). (F) Serum aspartate aminotransferase (AST), alanine aminotransferase (ALT), triglyceride and nonesterified fatty acid (NEFA) levels (*n* = 6). Data are represented as the mean ± SD values. Data were analysed using paired *t*‐test. ND, normal diet. *P < 0.05, **P < 0.01, ***P < 0.001 and ****P < 0.0001.

Seven‐week‐old *db/db* male mice were purchased from Shimizu Laboratory Supplies (Kyoto, Japan) and maintained in a pathogen‐free controlled environment. Littermate mice, born in the Shimizu Laboratory Supplies, were used in the experiments. The mice, housed in individual cages, were fed a normal diet (ND; 345 kcal/100 g, fat kcal 4.6%; CLEA, Tokyo, Japan) for 8 weeks, starting at 8 weeks of age, and an equal amount of feed was supplied for pair‐feeding. The sample size was analysed using EZR with the relative grip strength as a guide. With mean difference between the two groups being 1.02, the mean standard deviation being 0.44, the significance level set at 0.05 and the power calculated at 80%, the required sample size was six. Therefore, the sample size was set to six. Six mice were assigned to the following two groups: (1) mice fed without cow milk and (2) mice fed cow milk (Meiji Tokusen Hokkaido Gyunyu, Meiji Co., Ltd., Tokyo, Japan). In our study, the prevalence of sarcopenia was lower in elderly patients with type 2 diabetes who drank more than 150 mL/day of milk (*Figure* *S1*). Therefore, for approximately 150 mL/day (60 kg of body weight equivalent), 100 μL/day (40 g of body weight equivalent) of milk was administered to mice with a sonde for 8 weeks. Thereafter, the mice were fasted overnight and euthanized at 16 weeks of age by exposure to anaesthesia (4.0 mg/kg of midazolam, 0.3 mg/kg of medetomidine and 5.0 mg/kg of butorphanol) (*Figure* *1* *A*).

#### Stage 2

FMT has been broadly acknowledged as an approach to uncover the causal role of the gut microbiota in disease models related to the gut microbiota. Methods are described in detail in the supporting information.

### Measurement of momentum

Mice were individually housed in a cage with a running wheel (MK‐713; Muromachi Kikai). The number of rotations of the running wheel placed in each cage was recorded during each 12‐h night cycle using the software (CompACT AMS Version 3; Muromachi Kikai) in the computer connected to the running wheels. The momentum was measured for 5 days prior to sacrifice.

### Analytical procedures and glucose and insulin tolerance tests

Fifteen‐week‐old mice were subjected to intraperitoneal glucose tolerance testing (iPGTT) (2 g/kg of body weight) after a 16‐h fast and to insulin tolerance testing (ITT) (0.5 U/kg of body weight) after a 5‐h fast. Methods are described in detail in the supporting information.

### Measurement of grip strength

Grip strength was measured using a grip strength metre for mice (model DS2‐50N, IMADA Co., Ltd, Toyohashi, Japan) in another batch of 16‐week‐old mice. Six consecutive measurements were performed each day at 1‐min intervals. The investigators were blinded to the grouping of the mice. Grip strength was normalized to the body weight.

### Blood biochemistry

Blood samples were taken from fasted mice by cardiac puncture during euthanasia, and the serum samples were collected after centrifugation at 14 000 rpm for 10 min at 4°C. Methods are described in detail in the supporting information.

### Histological analysis of the soleus muscle

Soleus muscle tissue was obtained from the euthanized mice, fixed in 10% buffered formaldehyde and embedded in paraffin. Muscle sections were prepared and stained with haematoxylin and eosin (HE). The muscles were cut at the maximum bulge. Images were captured and cross‐sectional areas and diameters were measured using a BZ‐X710 fluorescence microscope (Keyence, Osaka, Japan).

### Gene expression analysis in murine muscle

The plantaris muscle of mice fasted for 16 h was excised and immediately frozen in liquid nitrogen. Methods are described in detail in the supporting information.

### Histological analysis of the jejunum and colon

Jejunum and colon removed from mice were immediately fixed in 10% buffered formaldehyde for 24 h at 22°C, embedded in paraffin, cut into 4‐μm‐thick sections and stained with HE and periodic acid Schiff (PAS) stain in Carnoy's solution. Methods are described in detail in the supporting information.

### mRNA microarray analysis of the jejunum

The jejunum of the mice fasted for 16 h was excised and immediately frozen in liquid nitrogen. Methods are described in detail in the supporting information. The global mRNA expression related to amino acids, fatty acids and glucose transporters was visualized using a volcano plot and heat map.

### Isolation of mononuclear cells from the small intestine of mice

Intestinal lamina propria (LPL) mononuclear cells were isolated using the Lamina Propria Dissociation Kit (130‐097‐410; Miltenyi Biotec, Germany), following the manufacturer's instructions. Methods are described in detail in the supporting information.

### Tissue preparation and flow cytometry

The cell suspension was obtained as described in the previous section. Methods are described in detail in the supporting information.

### Measurement of amino acid and organic acid levels in the serum and skeletal muscle samples and short‐chain fatty acid levels in the serum and faecal samples

The composition of amino acids and organic acids in murine sera and gastrocnemius muscle and that of short‐chain fatty acids (SCFAs) in murine sera and faeces were determined using gas chromatography–mass spectrometry (GC/MS) performed on an Agilent 7890B/7000D system (Agilent Technologies, Santa Clara, CA, USA). Methods are described in detail in the supporting information.

### 16S rRNA sequencing

A QIAamp DNA Faeces Mini Kit (Qiagen, Venlo, the Netherlands) was used to extract microbial DNA from frozen appendicular faecal samples, according to the manufacturer's instructions. Methods are described in detail in the supporting information.

### Statistical analysis

The data were analysed using the JMP Version 14.0 software (SAS, Cary, NC, USA). The paired *t*‐test was used to compare the two groups. One‐way analysis of variance (ANOVA) with the Holm–Šídák multiple‐comparison test was used to compare the four groups. Statistical significance was set at *P* < 0.05. Figures were generated using the GraphPad Prism software (Version 9.3.1; San Diego, CA, USA).

## Results

### Body weight, momentum, intraperitoneal glucose tolerance testing and insulin tolerance testing, hepatic enzymes and lipid metabolism in mice fed normal diet with or without milk

No difference was observed in the body weight of mice fed ND with milk (Milk+ group) and mice fed ND without milk (Milk− group) (*Figure* *1* *B*). The number of rotations of the running wheel significantly increased in the Milk+ group (*Figure* *1* *C*). Glucose tolerance was assessed using the iPGTT and ITT. No significant difference in the blood glucose levels measured with iPGTT was observed between the two groups (*Figure* *1* *D*). However, the ITT results revealed lower blood glucose levels in the Milk+ group (*Figure* *1* *D*). Absolute and relative grip strength were significantly increased in the Milk+ group compared with those in the Milk− group (*Figure* *1* *E*).

No significant difference in aspartate aminotransferase (AST), alanine aminotransferase (ALT) and triglyceride (TG) levels was observed between the two groups, whereas nonesterified fatty acid (NEFA) levels were significantly lower in the Milk+ group than in the Milk− group (*Figure* *1* *F*).

### Evaluation of soleus and plantaris muscles and expression of muscle atrophy genes in mice fed normal diet with and without milk

**Figure 2:**
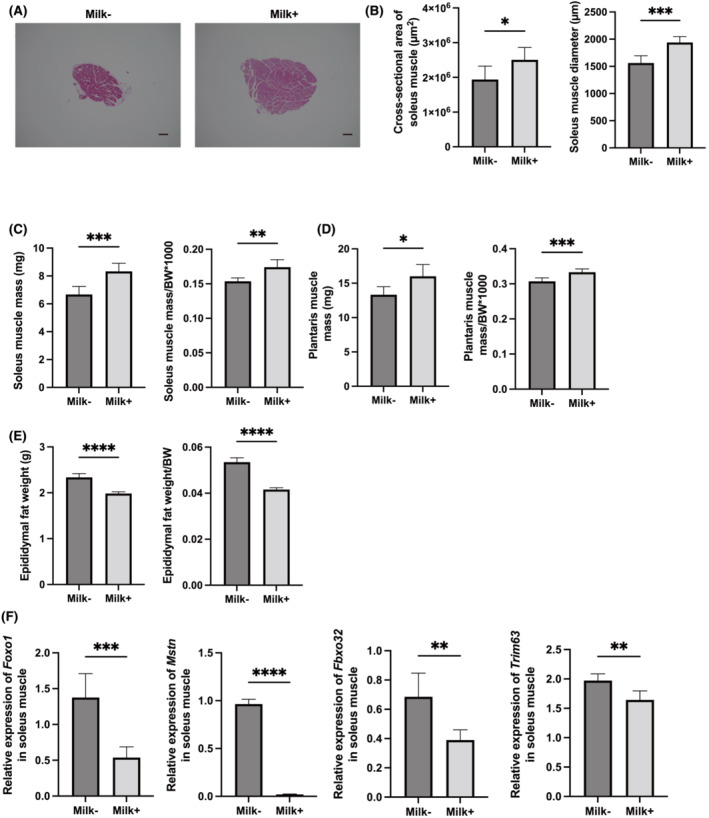
Administration of milk increased skeletal muscle mass and decreased the expression of genes related with muscle atrophy. (A) Representative images of haematoxylin and eosin‐stained soleus muscle sections. Soleus muscle tissues were collected at 16 weeks of age. The scale bar shows 100 μm. (B) Cross‐sectional area and diameter of soleus muscle (*n* = 6). (C) Absolute and relative soleus muscle weight, (D) absolute and relative plantaris muscle weight and (E) absolute and relative epididymal fat weight in 16‐week‐old mice (*n* = 6 in each case). (F) Relative mRNA expression of *Foxo1*, *Mstn*, *Fbxo32* and *Trim63* in the plantaris muscle normalized to the expression of *Gapdh* (*n* = 6). Data are represented as the mean ± SD values. Data were analysed using paired *t*‐test. BW, body weight. *P < 0.05, **P < 0.01, ***P < 0.001 and ****P < 0.0001.

Representative images of HE‐stained soleus muscle sections are shown in *Figure* *2* *A*. The cross‐sectional area of the soleus muscle was larger in the Milk+ group than that in the Milk− group (*Figure* *2* *B*). Absolute and relative soleus and plantaris muscle weights were significantly increased in the Milk+ group (*Figure* *2* *C,D*). The absolute and relative epididymal fat weights in the Milk+ group were lower than those in the Milk− group (*Figure* *2* *E*).

The expression of genes related to atrophy of the soleus muscle was determined. The expression of *Foxo1*, *Mstn*, *Fbxo32* and *Trim63* was significantly lower in the Milk+ group than in the Milk− group (*Figure* *2* *F*).

### Histological analysis of the jejunum and colon; ratio of ILC1s, ILC3s and M1 macrophages; and fold change in the expression of transporters in mice fed normal diet with and without milk

**Figure 3:**
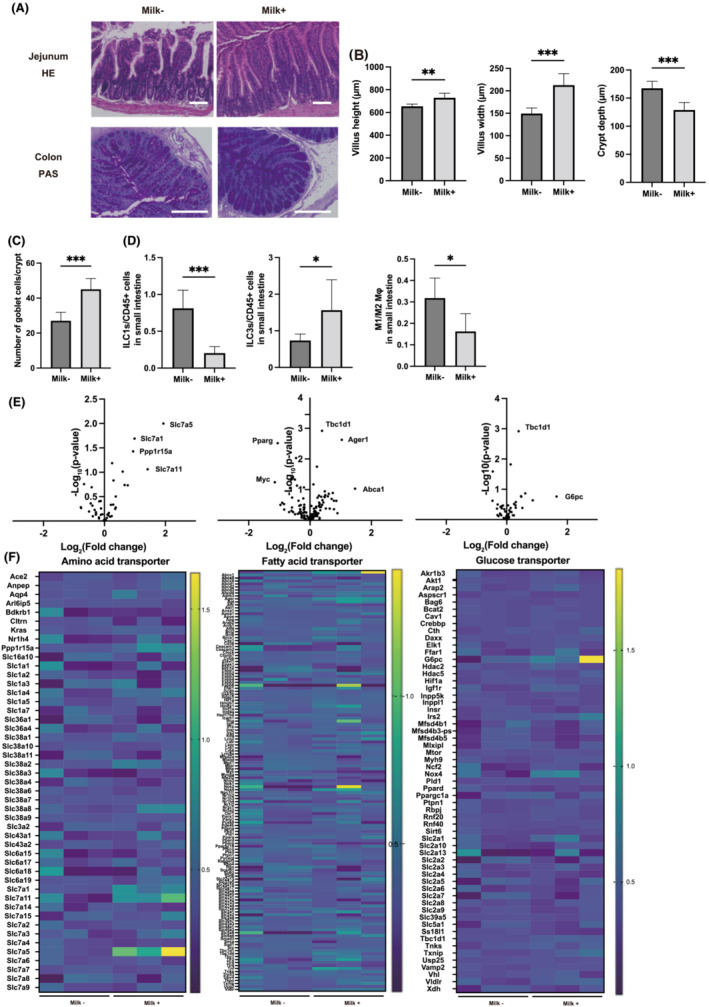
Administration of milk improved the inflammation in the intestine. (A) Representative images of haematoxylin and eosin (HE)‐stained jejunum and periodic acid Schiff (PAS)‐stained colon sections. Jejunum and colon tissues were collected at 16 weeks of age. The scale bar shows 100 μm. (B) Villus height and width, and crypt depth (*n* = 6). (C) Total goblet cells/crypt (*n* = 6). (D) Percentages of ILC1s to CD45‐positive cells and ILC3s to CD45‐positive cells, and ratio of M1 macrophages to M2 macrophages in the small intestine (*n* = 6 in each case). (E) Global mRNA expression related with amino acid transporter, fatty acid transporter and glucose transporter was visualized as a volcano plot (*n* = 3). (F) Global mRNA expression related with amino acid transporter, fatty acid transporter and glucose transporter was visualized as a heatmap (*n* = 3). Data are represented as the mean ± SD values. Data were analysed using paired *t*‐test. *P < 0.05, **P < 0.01 and ***P < 0.001.

Representative images of the jejunum and colon sections stained with HE and PAS are shown in *Figure* *3* *A*. A significant increase in villus weight and width was observed in the Milk+ group compared with that in the Milk− group (*Figure* *3* *B*). Additionally, a significant decrease in crypt depth and an increase in the ratio of goblet cells to crypts were observed in the Milk+ group (*Figure* *3* *C*).

The ratio of ILC1s and ILC3s to CD45‐positive cells and that of M1 macrophages to M2 macrophages in the small intestine were assessed. The number of ILC1 was significantly decreased in the Milk+ group, whereas that of ILC3 was increased (*Figure* *3* *D*). The M1/M2 ratio was lower in the Milk+ group than that in the Milk− group (*Figure* *3* *D*).

The differences in the expression of several transporters in skeletal muscle were determined using microarray analysis. In particular, amino acid transporter genes, such as *SIc7a5*, *SIc7a1*, *Ppp1r15a* and *SIc7a11*, had higher expression levels in the Milk+ group than those in the Milk− group. Fatty acid transporter genes, such as *Tbc1d1*, *Ager1* and *Abca1*, had higher expression levels in the Milk+ group, whereas *Pparg* and *Myc* had lower expression levels, than those in the Milk− group. Glucose transporter genes, such as *Tbc1d1* and *G6pc*, showed higher expression levels in the Milk+ group than those in the Milk− group (*Figure* *3* *E,F*).

### Analysis of amino acids and organic acids in the serum and skeletal muscle samples and short‐chain fatty acids in the serum and faecal samples of mice fed normal diet with or without milk

Next, the concentrations of amino acids and organic acids in the sera and skeletal muscle were investigated using a GC/MS system. The concentrations of valine, leucine, isoleucine, glycine, serine, threonine, methionine, phenylalanine, citric acid and lysine in the sera of the Milk+ group were significantly higher than those in the Milk− group (*Table* *1*). Similarly, the concentrations of valine, leucine, isoleucine, glycine, serine, aspartic acid, methionine, phenylalanine, citric acid and lysine in the skeletal muscle of the Milk+ group were significantly higher than those in the Milk− group. On the contrary, the concentration of succinic acid in the sera of the mice fed milk was higher than that in the Milk− group, whereas the opposite result was observed in the skeletal muscle.

**Table 1:** Amino acids and organic acids in sera and skeletal muscle, and short‐chain fatty acids in sera and faeces

Amino acids	Sera (μmol/L)			Skeletal muscle (μmol/mg)		
	Milk−	Milk+	*P*‐value	Milk−	Milk+	*P*‐value
Alanine	176.8 (58.9)	110.5 (50.5)	0.139	3.2 (1.0)	2.2 (0.4)	0.057
Valine	114.4 (92.2)	1003.7 (434.2)	0.002	0.0 (0.0)	0.9 (0.7)	0.038
Leucine	168.5 (25.5)	594.6 (162.4)	0.001	0.2 (0.0)	0.5 (0.1)	0.001
Isoleucine	333.8 (13.1)	363.2 (22.2)	0.046	2.3 (0.2)	2.8 (0.2)	0.027
Glycine	67.6 (7.8)	83.4 (9.6)	0.027	0.5 (0.1)	1.5 (0.4)	0.003
Serine	30.5 (20.3)	64.7 (10.9)	0.008	0.2 (0.1)	0.5 (0.1)	0.026
Threonine	55.6 (17.2)	116.1 (15.1)	0.012	0.3 (0.0)	0.4 (0.1)	0.111
Aspartic acid	31.4 (5.9)	31.0 (7.5)	0.933	0.5 (0.0)	0.6 (0.1)	0.010
Methionine	363.3 (12.5)	424.8 (11.0)	0.011	3.3 (0.1)	4.7 (0.6)	0.045
Phenylalanine	6.5 (15.1)	53.0 (12.9)	0.001	2.1 (0.6)	3.6 (1.0)	0.025
Lysine	48.2 (22.5)	96.6 (16.5)	0.017	0.3 (0.0)	0.4 (0.0)	0.015

Organic acids	Sera (μmol/L)			Skeletal muscle (μmol/mg)		
	Milk−	Milk+	*P*‐value	Milk−	Milk+	*P*‐value
Oxalic acid	391.3 (44.5)	454.9 (65.6)	0.092	2.6 (0.2)	2.7 (0.3)	0.503
Malonic acid	23.7 (19.7)	21.2 (8.3)	0.749	0.2 (0.2)	0.2 (0.2)	0.956
Succinic acid	57.2 (7.4)	46.7 (8.2)	0.013	0.2 (0.0)	0.3 (0.1)	0.010
Citric acid	93.5 (4.3)	103.1 (6.2)	0.034	0.1 (0.0)	0.3 (0.1)	0.004

Short‐chain fatty acids	Sera (μmol/L)			Faeces (μmol/mg)		
	Milk−	Milk+	*P*‐value	Milk−	Milk+	*P*‐value
Acetic acid	279.0 (42.8)	307.2 (59.8)	0.369	5.6 (1.2)	8.2 (2.2)	0.029
Propanoic acid	4.8 (0.5)	213.6 (25.7)	<0.001	14.2 (1.4)	18.9 (3.2)	0.009
Butanoic acid	65.4 (5.0)	109.6 (19.0)	0.037	14.6 (0.3)	41.2 (9.9)	<0.001

*Note*: Data are expressed as mean (SD). Student's paired *t*‐test was conducted between the two groups.

The concentrations of SCFAs in the serum and faecal samples were measured. The concentration of acetic acid in the sera was not different between the two groups, whereas that of propanoic acid and butanoic acid in the Milk+ group was significantly higher than that in the Milk− group (*Table* *1*). In addition, the concentrations of acetic acid, propanoic acid and butanoic acid in the skeletal muscle of the Milk+ group were higher than those in the Milk− group.

### Faecal microbiota transplantation from mice donors fed normal diet with or without milk to mice fed normal diet without milk

**Figure 4:**
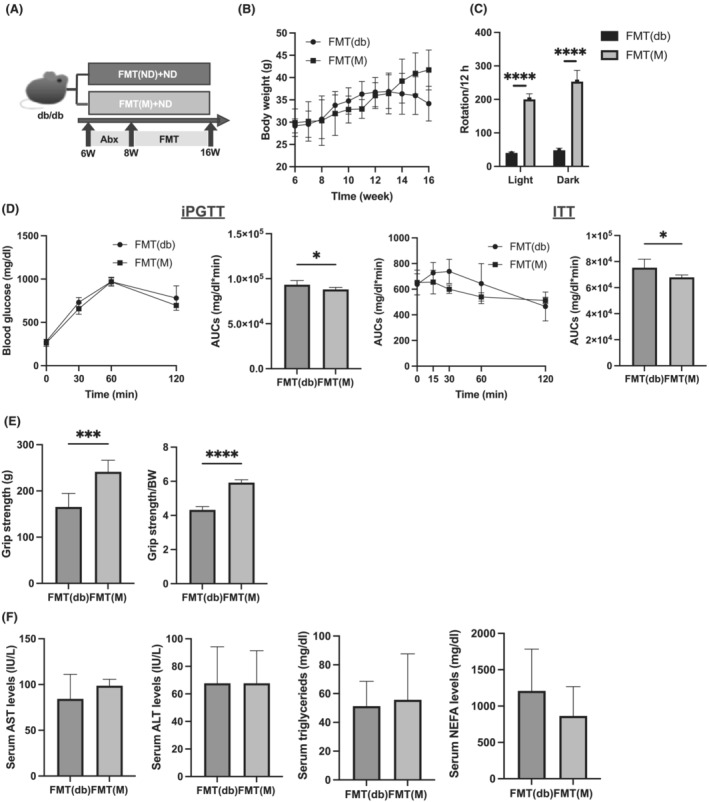
Faecal microbiota transplantation (FMT) of *db/db* mice fed normal diet (ND) with milk increased grip strength. (A) Depletion of recipient gut microbiota by antibiotics was performed for 2 weeks from 6 to 8 weeks of age prior to FMT. After 3 days of recovery, FMT was performed twice weekly; 200–300 mg of fresh stool was collected from 16‐week‐old *db/db* mice fed ND with milk and those without milk, respectively. (B) Changes in the body weight (*n* = 6). (C) The numbers of rotation in the light and dark phases using the running wheel (*n* = 6). (D) Results of intraperitoneal glucose tolerance testing (iPGTT; 2 g/kg of body weight) for 15‐week‐old mice and the area under the curve (AUC) analysis (*n* = 6). Results of insulin tolerance testing (ITT; 0.5 U/kg of body weight) for 15‐week‐old mice and the AUC analysis (*n* = 6). (E) Absolute and relative grip strength (*n* = 6). (F) Serum aspartate aminotransferase (AST), alanine aminotransferase (ALT), triglyceride and nonesterified fatty acid (NEFA) levels (*n* = 6). Data are represented as the mean ± SD values. Data were analysed using paired *t*‐test. ***P < 0.001 and ****P < 0.0001.

FMT was performed to uncover the role of the gut microbiota and the effect of milk supplementation with diet (*Figure* *4* *A*). No difference was observed in body weight between the FMT from the Milk− group (FMT(db) group) and FMT from the Milk+ group (FMT(M) group) (*Figure* *4* *B*). The number of rotations on the running wheel was significantly higher in the FMT(M) group than that in the FMT(db) group (*Figure* *4* *C*). Moreover, the areas under the curve (AUCs) of iPGTT and ITT in the FMT(db) group were lower than those in the FMT(M) group (*Figure* *4* *D*). Absolute and relative grip strength were significantly higher in FMT(M) mice than those in FMT(db) mice (*Figure* *4* *E*). No differences were observed in the levels of AST, ALT, TG and NEFA between the two groups (*Figure* *4* *F*).

### Evaluation of soleus and plantaris muscles and expression of muscle atrophy genes in FMT(db) and FMT(M) mice

**Figure 5:**
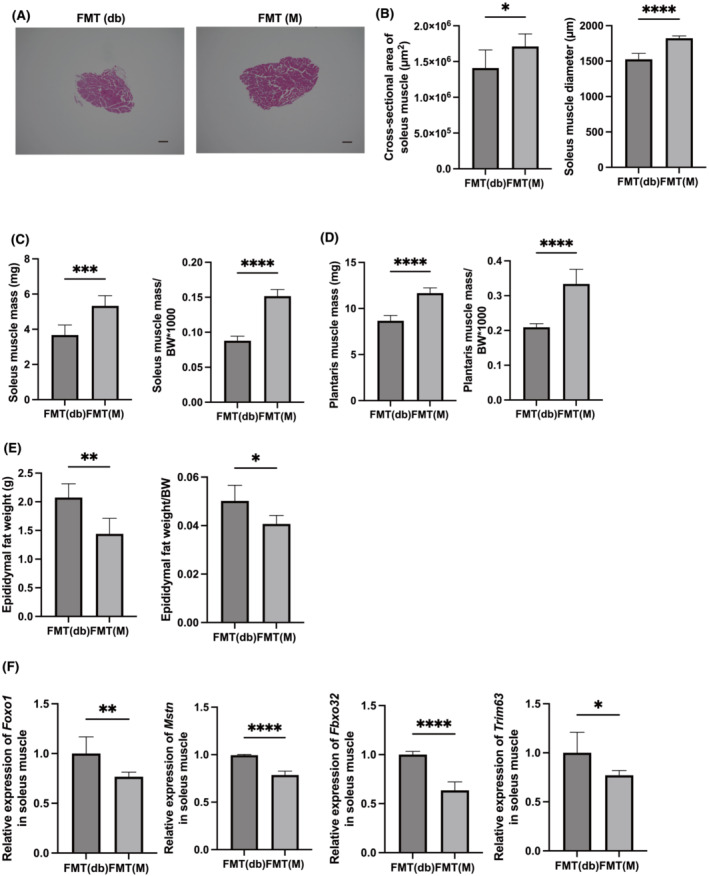
Faecal microbiota transplantation (FMT) of *db/db* mice fed normal diet with milk increased skeletal muscle mass and decreased the expression of genes related with muscle atrophy. (A) Representative images of haematoxylin and eosin‐stained soleus muscle sections. Soleus muscle tissues were collected at 16 weeks of age. The scale bar shows 100 μm. (B) Cross‐sectional area and diameter of soleus muscle (*n* = 6). (C) Absolute and relative soleus muscle weight, (D) absolute and relative plantaris muscle weight and (E) absolute and relative epididymal fat weight in 16‐week‐old mice (*n* = 6 in each case). (F) Relative mRNA expression of *Foxo1*, *Mstn*, *Fbxo32* and *Trim63* in the plantaris muscle normalized to the expression of *Gapdh* (*n* = 6). Data are represented as the mean ± SD values. Data were analysed using paired *t*‐test. BW, body weight. *P < 0.05, **P < 0.01, ***P < 0.001 and ****P < 0.0001.

Representative images of HE‐stained soleus muscle sections are shown in *Figure* *5* *A*. The cross‐sectional area of the soleus muscle was higher in the FMT(M) group than that in the FMT(db) group (*Figure* *5* *B*). The absolute and relative weights of the soleus and plantaris muscles significantly increased in the FMT(M) group (*Figure* *5* *C,D*). Moreover, the absolute and relative epididymal fat weights in the FMT(M) group were lower than those in the FMT(db) group (*Figure* *5* *E*). The expression of muscle atrophy‐related genes, such as *Foxo1*, *Mstn*, *Fbxo32* and *Trim63*, in the soleus muscle was decreased in the FMT(M) group (*Figure* *5* *F*).

### Analysis of amino acids and organic acids in the serum and skeletal muscle samples and short‐chain fatty acids in the serum and faecal samples of the FMT(db) and FMT(M) groups

The serum concentrations of valine, leucine, isoleucine, glycine, serine, threonine, methionine, phenylalanine and lysine in the FMT(M) group were significantly higher than those in the FMT(db) group (*Table* *2*). Similarly, the concentrations of valine, leucine, isoleucine, glycine, methionine, phenylalanine, citric acid and lysine in the skeletal muscle of the FMT(M) group were significantly higher than those in the FMT(db) group. The results were similar to those observed in the Milk− and Milk+ groups. In contrast, the opposite trend observed for succinate concentration in the serum and muscle in the previous experiment was not observed in the FMT groups.

**Table 2:** Amino acids and organic acids in sera and skeletal muscle, and short‐chain fatty acids in sera and faeces

Amino acids	Sera (μmol/L)			Skeletal muscle (μmol/mg)		
	FMT(db)	FMT(M)	*P*‐value	FMT(db)	FMT(M)	*P*‐value
Alanine	507.2 (412.2)	683.5 (594.9)	0.576	3.7 (0.8)	4.3 (0.3)	0.146
Valine	479.0 (20.0)	691.2 (143.5)	0.006	0.5 (0.0)	0.7 (0.2)	0.018
Leucine	336.9 (5.8)	482.3 (131.)	0.023	0.3 (0.0)	0.4 (0.1)	0.003
Isoleucine	210.4 (15.7)	301.7 (88.7)	0.034	0.2 (0.0)	0.3 (0.0)	<0.001
Glycine	89.9 (3.5)	170.1 (4.8)	<0.001	1.9 (0.1)	2.3 (0.2)	0.001
Serine	51.7 (1.9)	91.8 (6.6)	<0.001	0.3 (0.0)	0.4 (0.0)	0.138
Threonine	64.6 (7.7)	166.1 (0.6)	<0.001	0.3 (0.0)	0.2 (0.0)	0.586
Aspartic acid	26.7 (8.2)	30.4 (13.2)	0.585	0.1 (0.0)	0.1 (0.0)	0.197
Methionine	237.1 (36.8)	480. (2.8)	0.001	7.2 (0.4)	8.5 (0.6)	0.008
Phenylalanine	81.3 (2.9)	146.4 (5.5)	<0.001	1.7 (0.0)	2.0 (0.1)	<0.001
Lysine	53.3 (2.3)	237.8 (14.9)	<0.001	1.9 (0.1)	3.3 (0.5)	0.001

Organic acids	Sera (μmol/L)			Skeletal muscle (μmol/mg)		
	Milk−	Milk+	*P*‐value	Milk−	Milk+	*P*‐value
Oxalic acid	11.2 (1.0)	12. (2.1)	0.423	0.1 (0.0)	0.1 (0.0)	0.410
Malonic acid	8.2 (1.1)	7.8 (1.1)	0.590	0.1 (0.0)	0.0 (0.0)	0.534
Succinic acid	8.6 (3.2)	10.1 (6.4)	0.622	0.2 (0.0)	0.3 (0.1)	0.042
Citric acid	148.9 (21.5)	164.4 (39.1)	0.426	0.1 (0.0)	0.2 (0.1)	0.005

Short‐chain fatty acids	Sera (μmol/L)			Faeces (μmol/mg)		
	FMT(db)	FMT(M)	*P*‐value	FMT(db)	FMT(M)	*P*‐value
Acetic acid	242.4 (26.7)	343.8 (43.9)	<0.001	4.2 (1.5)	7.3 (0.9)	0.002
Propanoic acid	4.5 (0.2)	64.9 (3.2)	<0.001	9.5 (1.7)	16.0 (6.2)	0.033
Butanoic acid	20.9 (1.0)	31.3 (1.6)	0.037	6.3 (1.0)	42.1 (22.4)	0.003

*Note*: Data are expressed as mean (SD). Student's paired *t*‐test was conducted between the two groups. Abbreviation: FMT, faecal microbiota transplantation.

The concentrations of SCFAs in the sera and faeces were determined. The concentrations of acetic, propanoic and butanoic acids were significantly higher in the FMT(M) group than those in the FMT(db) group (*Table* *2*).

### Microbiota composition in the Milk−, Milk+, FMT(db) and FMT(M) groups

**Figure 6:**
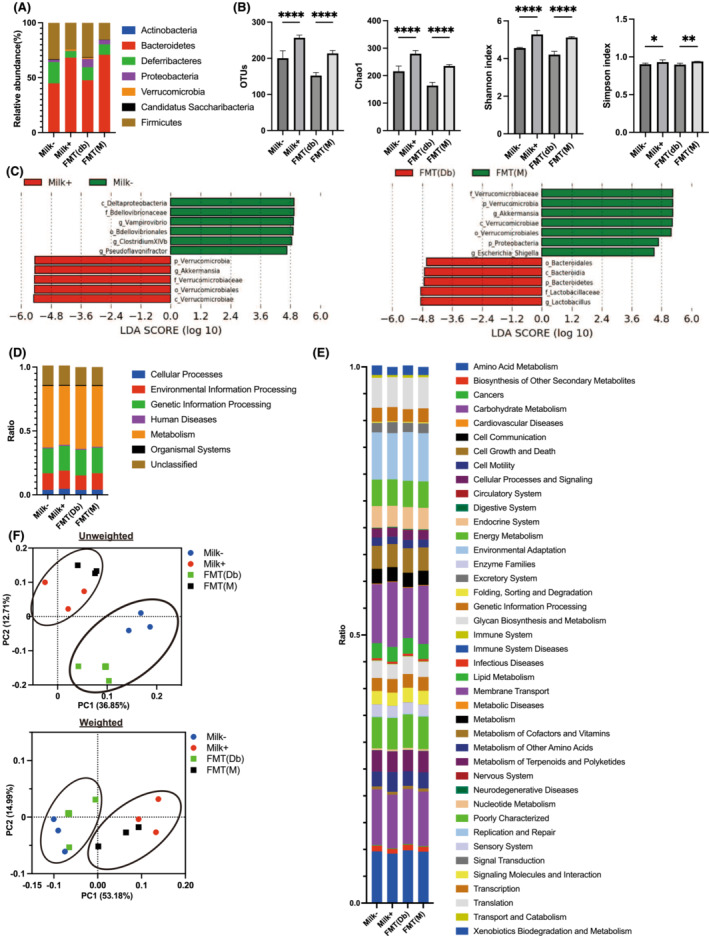
Components of the gut microbiota. (A) Relative abundance of gut microbiota at the phylum levels (*n* = 3). (B) Operational taxonomic units (OTUs) (*n* = 3), Chao1 (*n* = 3), Shannon index (*n* = 3) and Simpson index (*n* = 3). (C) Linear discriminant analysis (LDA) scores of gut microbiota of the Milk− (green) and Milk+ (red) groups and the FMT(db) (green) and FMT(M) (red) groups. (D) Description of Kyoto Encyclopedia of Genes and Genomes (KEGG) pathway class I (*n* = 3). (E) Description of KEGG pathway class II (*n* = 3). (F) Unweighted principal coordinate analysis (PCoA) plots and k‐means clustering for gut microbiota and weighted PCoA plots and k‐means clustering for gut microbiota. Data are the mean ± SD values. Data were analysed using one‐way analysis of variance with the Holm–Šídák multiple‐comparison test. FMT, faecal microbiota transplantation. *P < 0.05, **P < 0.01, ***P < 0.001 and ****P < 0.0001.

The relative abundance of different phyla in all four groups was investigated. A higher abundance of phyla was observed in the Milk+ and FMT(M) groups than that in the Milk− and FMT(db) groups, and a lower abundance of the phylum *Firmicutes* was observed (*Figure* *6* *A*). The number of operational taxonomic units (OTUs) was increased in the Milk+ and FMT(M) groups (*Figure* *6* *B*). Chao1, Shannon index and Simpson index, the three indicators of diversity, were higher in the Milk+ and FMT(M) groups than those in the Milk− and FMT(db) groups (*Figure* *6* *B*).

The linear discriminant analysis effect size (LEfSe) algorithm was employed to identify the specific taxa that were variably distributed between the Milk− and Milk+ groups and between the FMT(db) and FMT(M) groups. Six taxa were over‐represented (including the phylum *Verrucomicrobia* and the genus *Akkermansia*), and five taxa were under‐represented (including the class *Deltaproteobacteria* and the family *Bdellovibrionaceae*) in the Milk+ group compared with those in the Milk− group (*Figure* *6* *C*). Seven taxa were over‐represented (including the phylum *Verrucomicrobia* and the genus *Akkermansia*), and five taxa were under‐represented (including the family *Lactobacillaceae* and the phylum *Bacteroides*) in the FMT(M) group compared with those in the FMT(db) group (*Figure* *6* *C*).

Next, PICRUSt was used to analyse the differences in Kyoto Encyclopedia of Genes and Genomes (KEGG) pathways of gut microbiota between the Milk− and Milk+ groups and between the FMT(db) and FMT(M) groups. Among the KEGG pathway class I, the pathways of cellular processes and environmental information processing were upregulated, and those of metabolism and organismal systems were downregulated in the Milk+ group compared with those in the Milk− group. In contrast, the pathways of environmental information processing in the FMT(M) group were upregulated, and those of human diseases, metabolism and organismal systems were downregulated compared with those in the FMT(db) group (*Table* *S2* and *Figure* *6* *D*). Among the KEGG pathway class II, the pathways of cell motility, genetic information processing, membrane transport, signal transduction and transcription were upregulated, and those of amino acid metabolism, biosynthesis of other secondary metabolites, carbohydrate metabolism, cellular processes and signalling, enzyme families, glycan biosynthesis and metabolism, metabolism of other amino acids, nucleotide metabolism, replication and repair, and transport and catabolism were downregulated in the Milk+ group compared with those in the Milk− group. The pathways of membrane transport and transcription were upregulated, and those of biosynthesis of other secondary metabolites, endocrine system, energy metabolism, folding, sorting and degradation, and glycan biosynthesis and metabolism were downregulated in the FMT(M) group compared with those in the FMT(db) group (*Table* *S3* and *Figure* *6* *E*).

The effectiveness of FMT was determined using principal coordinate analysis (PCoA). The unweighted and weighted clustering results showed that the Milk− and FMT(db) groups and the Milk+ and FMT(M) groups belonged to the same cluster (*Figure* *6* *F*).

## Discussion

In this study, administration of milk to model mice of sarcopenic obesity increased muscle strength and muscle mass and decreased visceral fat mass, resulting in a significant increase in physical activity. Furthermore, transplantation of faecal matter from the Milk+ group not only improved sarcopenia obesity but also significantly improved glucose intolerance. This suggests that not only nutrient supplementation but also modification of the gut microbiota by the administration of milk might be involved in the improvement of sarcopenic obesity.

Amino acid and organic acid concentrations in the skeletal muscle were increased in the Milk+ group. Milk contains many amino acids, including the essential ones. The concentrations of these amino acids in both serum and skeletal muscle samples were higher in the Milk+ group than those in the Milk− group. Besides the increased nutrient intake, increased amino acid absorption from the small intestine was also the reason behind the higher concentration of amino acids in the skeletal muscle in the Milk+ group. The expression of the amino acid absorption transporter genes, *Slc7a1*, *Slc7a5* and *Slc7a11*, was significantly elevated in the Milk+ group. These results suggested that milk improves intestinal inflammation. The results of flow cytometry showed that the number of ILC3, which secretes IL22 in the intestinal tract and promotes mucin secretion from goblet cells,^14^ was increased in the lamina propria of the small intestine in the Milk+ group. Histological analyses showed that milk administration improved villus atrophy and thickened the mucin layer. Inflammatory cells, such as ILC1 and M1 macrophages, in the lamina propria of the small intestine were decreased in the Milk+ group.^15^ The production of SCFAs, including butyric acid, was increased in the faeces of the Milk+ group. Considering the fact that SCFAs have been reported to enhance the secretion of IL22 from ILC3,^16^ the increase in SCFAs in the intestine upon milk administration could be one of the anti‐inflammatory mechanisms induced by milk. Several previous reports have indicated that intestinal inflammation can cause malabsorption of nutrients, such as amino acids, which are important for stimulating muscle biosynthesis.^17^ Interestingly, the results of the KEGG pathway revealed that the flora involved in amino acid metabolism was reduced in the Milk+ and FMT(M) groups. This suggests that the increase in amino acid absorption due to milk intake might be related more to the increase in the expression of amino acid absorption transporters via anti‐inflammatory effects than to changes in the gut microbiota. In summary, the increase in the number of anti‐inflammatory cells due to the increase in the concentration of SCFAs in the gut following milk administration and the decrease in the number of inflammatory cells increased the absorption of amino acids from the small intestine, which might have increased amino acid concentrations in the blood and skeletal muscle and improved sarcopenia.

We examined the modification of the gut microbiota upon administration of milk and FMT from the Milk+ group. The gut microbiota plays an important role in nutrient absorption, metabolic homeostasis, protection against infection and development of systemic and mucosal immunity.^18^ Dysbiosis of the gut microbiota has close pathological and physiological interactions with obesity and metabolic syndrome^19^; it is, therefore, relevant to investigate the gut microbiota to assess metabolic changes associated with diet. In the present study, the abundance of the genus *Akkermansia* was significantly increased in the faeces of the Milk+ and FMT(M) groups compared with that in the Milk− and FMT(db) groups. *Akkermansia* is a genus of anaerobic gram‐negative bacteria that is mucinolytic and is distributed in the digestive tracts of a wide range of vertebrates.^20^ Two species have been identified: *Akkermansia muciniphila* , isolated from human faeces,^21^ and *Akkermansia glycaniphila*, isolated from python faeces.^22^ Both are biochemically anaerobic and nonmotile oval and can use mucin as the sole carbon, nitrogen and energy source. However, in other studies in which the genome of *Akkermansia* species was examined using the 16S rRNA analysis, *A. glycaniphila* was not detected in the gut microbiota of mammals, including humans and mice.^23^ *A. muciniphila* produces mucinolytic enzymes in the mucus layer of the epithelium, can use mucin as a nitrogen and carbon source and degrades these substances to acetic and propanoic acids.^24^ The increase in SCFAs, such as acetic acid, propanoic acid and butanoic acid, observed in our study might be partly due to the increase in the number of *A. muciniphila* . In addition, *A. muciniphila* enrichment can be used as an indicator to evaluate the metabolic state of the body, such as glucose tolerance, serum lipids and fat cell distribution in humans.^25^ *A. muciniphila* enrichment has been reported to be low in the intestines of patients with type 2 diabetes.^26^ *A. muciniphila* enrichment is not only an indicator of metabolic syndrome, but it has also been reported that oral administration of *A. muciniphila* eliminated obesity induced by a high‐fat diet in animal experiments.^27^ An increase in the genus *Akkermansia* in the gut microbiota after milk administration has been reported.^28^ In the present study, a reduction in visceral fat mass in the Milk+ group was also observed in the FMT(M) group, suggesting that modification of the gut microbiota and an increase in the number of *A. muciniphila* might be involved in the reduction of visceral fat mass.

There are few reports on the association between muscle loss and *A. muciniphila* . An observational study in humans reported a decrease in the genus *Akkermansia* in the gut microbiota of sarcopenia patients with cirrhosis^29^ and an increase in the genus *Akkermansia* in the gut microbiota of sarcopenia patients with chronic kidney disease.^30^ In mouse models, moderate‐intensity exercise has shown significant and reproducible changes in the composition of the gut microbiota, such as the genus *Akkermansia*.^31^ In the present study, increased locomotion was observed in the mice fed milk and the mice subjected to FMT from mice fed milk. This is expected to be primarily due to the increased muscle mass and strength; increased physical activity may have contributed to the modification of the gut microbiota. Further studies are required in this regard.

Finally, we discuss the association between milk consumption and improvement in glucose intolerance. Several meta‐analyses have reported that milk intake is associated with glucose tolerance. A recent study of 147 812 individuals from 21 countries also showed that a higher intake of full‐fat dairy products is associated with a lower incidence of diabetes.^32^ In contrast, an observational study of 97 811 people in Denmark also reported that milk intake was not associated with a lower risk of type 2 diabetes or obesity.^33^ The UK Biobank study of 1 904 220 subjects also found no significant correlation between milk consumption and the development of type 2 diabetes.^34^ In this study, both groups were fed the same amount of food by pair‐feeding, but the mice fed milk received milk in addition to the food they were fed, resulting in a slight increase in total calorie intake compared with that in mice that were not fed milk. This might have affected the AUC of iPGTT in the Milk+ group, which tended to be lower than that in the Milk− group, but the difference was not statistically significant. On the contrary, the fact that glucose intolerance was clearly improved in the FMT(M) group compared with that in the FMT(db) group suggested that the mechanism of improvement in glucose intolerance by milk likely involves modification of the gut microbiota. There are several reports on the association of *A. muciniphila* with type 2 diabetes. In an animal study, it has been reported that administration of either pasteurized *A. muciniphila* or its outer membrane protein, Amuc_1100*, activated Toll‐like receptor 2, increased the expression of tight junction protein, restored high‐fat diet‐induced obesity and reduced insulin resistance.^35^ In human studies, a decreased abundance of *A. muciniphila* has also been observed in patients diagnosed with type 2 diabetes or prediabetes,^26^ and the relative proportion of *A. muciniphila* has been reported to be significantly negatively correlated with haemoglobin A1c levels.^36^ It is suggested that modification of the gut microbiota, including *Akkermansia* species, might contribute significantly to the improvement in glucose tolerance induced by milk.

In conclusion, the improvement of sarcopenic obesity and blood glucose levels in the mice fed ND with milk, which was also observed in mice with FMT from the mice fed ND with milk, suggests that not only increased intake of nutrients, such as amino acids, upon milk intake but also increased absorption of amino acids through the modification of genes related to amino acid transporters in the small intestinal epithelium via improvement of inflammation in the small intestine might contribute to the improvement of sarcopenic obesity by milk.

## Conflict of interest statement

Takuro Okamura declares that he has no conflict of interest. Masahide Hamaguchi has received grants from Asahi Kasei Pharma, Nippon Boehringer Ingelheim Co., Ltd., Mitsubishi Tanabe Pharma Corporation, Daiichi Sankyo Co., Ltd., Sanofi K.K., Takeda Pharmaceutical Company Limited, Astellas Pharma Inc., Kyowa Kirin Co., Ltd., Sumitomo Dainippon Pharma Co., Ltd., Novo Nordisk Pharma Ltd. and Eli Lilly Japan K.K., outside the submitted work. Hanako Nakajima, Nobuko Kitagawa and Saori Majima declare no conflict of interest. Takafumi Senmaru has received personal fees from Ono Pharma Co., Ltd., Mitsubishi Tanabe Pharma Co., Astellas Pharma Inc., Kyowa Hakko Kirin Co., Ltd., Sanofi K.K., MSD K.K., Kowa Pharma Co., Ltd., Taisho Toyama Pharma Co., Ltd., Takeda Pharma Co., Ltd., Kissei Pharma Co., Ltd., Novo Nordisk Pharma Ltd. and Eli Lilly Japan K.K., outside the submitted work. Emi Ushigome has received grants from the Japanese Study Group for Physiology and Management of Blood Pressure and the Astellas Foundation for Research on Metabolic Disorders (Grant Number 4024). Donated Fund Laboratory of Diabetes Therapeutics is an endowment department, supported with an unrestricted grant from Ono Pharmaceutical Co., Ltd., and received personal fees from AstraZeneca plc, Astellas Pharma Inc., Daiichi Sankyo Co., Ltd., Kyowa Hakko Kirin Company Ltd., Kowa Pharmaceutical Co., Ltd., MSD K.K., Mitsubishi Tanabe Pharma Corp., Novo Nordisk Pharma Ltd., Taisho Toyama Pharmaceutical Co., Ltd., Takeda Pharmaceutical Co., Ltd., Nippon Boehringer Ingelheim Co., Ltd., and Sumitomo Dainippon Pharma Co., Ltd., outside the submitted work. Naoko Nakanishi and Ryoichi Sasano declare no conflict of interest. Michiaki Fukui has received grants from Nippon Boehringer Ingelheim Co., Ltd., Kissei Pharma Co., Ltd., Mitsubishi Tanabe Pharma Co., Daiichi Sankyo Co., Ltd., Sanofi K.K., Takeda Pharma Co., Ltd., Astellas Pharma Inc., MSD K.K., Kyowa Hakko Kirin Co., Ltd., Sumitomo Dainippon Pharma Co., Ltd., Kowa Pharmaceutical Co., Ltd., Novo Nordisk Pharma Ltd., Ono Pharma Co., Ltd., Sanwa Kagaku Kenkyusho Co., Ltd., Eli Lilly Japan K.K., Taisho Pharma Co., Ltd., Terumo Co., Teijin Pharma Ltd., Nippon Chemiphar Co., Ltd., Johnson & Johnson K.K. Medical Co. and Abbott Japan Co., Ltd., and received personal fees from Nippon Boehringer Ingelheim Co., Ltd., Kissei Pharma Co., Ltd., Mitsubishi Tanabe Pharma Corp., Daiichi Sankyo Co., Ltd., Sanofi K.K., Takeda Pharma Co., Ltd., Astellas Pharma Inc., MSD K.K., Kyowa Kirin Co., Ltd., Sumitomo Dainippon Pharma Co., Ltd., Kowa Pharma Co., Ltd., Novo Nordisk Pharma Ltd., Ono Pharma Co., Ltd., Sanwa Kagaku Kenkyusho Co., Ltd., Eli Lilly Japan K.K., Taisho Pharma Co., Ltd., Bayer Yakuhin, Ltd., AstraZeneca K.K., Mochida Pharma Co., Ltd., Abbott Japan Co., Ltd., Medtronic Japan Co., Ltd., Arkley Inc., Teijin Pharma Ltd. and Nipro Corp., outside the submitted work.

## Supporting information


**Data S1.** Supporting information

**Table S1.** Primer sequences
**Table S2.** KEGG pathway in four groups
**Table S3.** KEGG pathway Class II in four groups

**Figure S1.** Strategy for innate lymphoid cells (ILCs). Representative flow cytometry plots of liver CD45+ Live & Dead‐ Lin‐ CD127+ RORg‐ GATA‐3‐ T‐bet+ ILC1s, CD45+ Live & Dead‐ Lin‐ CD127+ RORg‐ GATA‐3+ ILC2s and CD45+ Live & Dead‐ Lin‐ CD127+ RORg+GATA‐3‐ ILC3s in each group at 16‐weeks of age.
**Figure S2.** Strategy for macrophages. Representative flow cytometry plots of liver CD45+ F4/80+ CD206‐ CD11c+ M1 macrophages and CD45+ F4/80+ CD206+ CD11c‐ M2 macrophages in each group at 16 weeks of age.
