# Supplementary material for: Milk protects against sarcopenic obesity due to increase in the genus Akkermansia in faeces of db/db mice
Source: J Cachexia Sarcopenia Muscle. 2023 May 2;14(3):1395–409. doi: 10.1002/jcsm.13245 (PMC10235896; doi:10.1002/jcsm.13245)
Supplement: Supplementary file 1 — Data S1. Supporting information [file JCSM-14-1395-s001.docx]

# Methods

*Mice*

Stage 2

FMT has been broadly acknowledged as an approach to uncover the causal role of the gut microbiota in disease models related to the gut microbiota. To achieve better engraftment, depletion of the recipient gut microbiota using antibiotics (ampicillin, neomycin, metronidazole: 1 g/L each; vancomycin: 0.5 g/L; 200 μL/day by oral-gastric gavage) was performed for 2 weeks from 6 to 8 weeks of age prior to FMT (S1). After 3 days of recovery, FMT was performed twice weekly. Briefly, 200–300 mg of fresh stool was collected from 16-week-old *db/db* mice fed without milk and from those fed milk. The stool was homogenized in 5 mL of phosphate-buffered saline (PBS) and sedimented under gravity for 2 min; 200 μL of the supernatant, thus obtained, was administered to each receiving mouse (S2) and used for extraction of the gut. Body weight changes, glucose tolerance, and grip strength were evaluated for *db/db* mice subjected to FMT of stool from 16-week-old *db/db* mice fed without milk (FMT(db)) and from *db/db* mice fed milk (FMT(M)). The mice were then sacrificed, and absolute and relative soleus and plantaris muscle mass and epididymal fat mass were determined. At 16-weeks of age, after overnight fasting, all mice were killed by administering a combination of anesthetics.

*Analytical procedures and glucose and insulin tolerance tests*

Fifteen-week-old mice were subjected to intraperitoneal glucose tolerance testing (iPGTT) (2 g/kg of body weight) after a 16 h fast and to insulin tolerance testing (ITT) (0.5 U/kg body weight) after a 5 h fast (S3). Blood samples were collected from the tail vein. Blood glucose levels were measured using a glucometer (Gultest mint II; Sanwa Kagaku Kenkyusho, Nagoya, Japan). iPGTT and ITT were performed in different mice. Blood glucose levels were monitored at 0, 15, 30, 60, and 120 min after injection. The area under the curve (AUC) of the iPGTT and ITT results was analyzed (*n* = 6).

*Blood biochemistry*

Blood samples were taken from fasted mice by cardiac puncture during euthanasia, and the serum samples were collected after centrifugation at 14,000 rpm for 10 min at 4 °C. The collected serum was stored at −30 °C until it was mailed to a subcontractor for analyses. Alanine aminotransferase (ALT) levels were measured using the standardized support method described by the Japanese Society for Clinical Chemistry (S4). Triglyceride (TG) (S5) and non-esterified fatty acid (NEFA) (S6) levels were measured using enzymatic methods (TG, GK-GPO, glycerol blanking method; NEFA, acyl-CoA synthetase- acyl-CoA oxidase-3-methyl-N-ethyl-N-(beta-hydroxyethyl)-aniline). Biochemical examinations (*n* = 6) were performed at the FUJIFILM Wako Pure 18 Chemical Corporation (Osaka, Japan).

*Gene expression analysis in murine muscle*

The plantaris muscle of mice fasted for 16 h was excised and immediately frozen in liquid nitrogen. The samples were homogenized in ice-cold QIAzol Lysis reagent (Qiagen, Venlo, The Netherlands) at 4000 rpm for 2 min in a ball mill, and total RNA was extracted according to the manufacturer’s instructions. A High-Capacity cDNA Reverse Transcription Kit (Applied Biosystems, Foster City, CA, USA) was used to reverse transcribe the total RNA (0.5 μg) to first-strand cDNA, according to the manufacturer’s instructions. The mRNA expression of *Foxo1*, *Mstn*, *Fbxo32*, and *Trim63* in the plantaris muscle was quantified using real-time reverse transcription-polymerase chain reaction (RT-PCR); TaqMan Fast Advanced Master Mix (Applied Biosystems) was used according to the manufacturer’s instructions. The PCR conditions were as follows: one cycle of 2 min at 50 °C and 20 s at 95 °C, followed by 40 cycles of 1 s at 95 °C and 20 s at 60 °C. The relative expression of each target gene was normalized to the *GAPDH* threshold cycle (Ct) values and quantified using the comparative threshold cycle 2^−∆∆Ct^ method. Signals from the ND-fed mice were assigned a relative value of 1.0. Expression levels in six mice from each group were determined, and RT-PCR was performed in triplicate for each sample (*n* = 6). Primer sequences for each gene are presented in Table S1.

*Histological analysis of the jejunum and colon*

Jejunum and colon removed from mice were immediately fixed in 10% buffered formaldehyde for 24 h at 22 °C, embedded in paraffin, cut into 4 µm-thick sections, and stained with HE and periodic acid Schiff (PAS) stain in Carnoy’s solution. Images of the stained sections were captured using a fluorescence microscope (BZ-X710; Keyence). The villus height/width and crypt depth were estimated using the HE-stained sections at five locations per slide for each group of 10 animals with the ImageJ software (Version 1.53 k, NIH, Bethesda, MD, USA). Mucin grains and goblet cells (PAS^+^) were enumerated and reported as the average number of goblet cells (PAS^+^) per 10 crypts using the ImageJ software, as reported previously (*n* = 6) (S7).

*mRNA microarray analysis of the jejunum*

The jejunum of the mice fasted for 16 h was excised and immediately frozen in liquid nitrogen. The RNA extraction method was the same as that for the muscle described in the section, “*Gene expression analysis in murine muscle*.” A cDNA library was constructed using the TruSeq^®^ Stranded mRNA kit (Qiagen, Carlsbad, CA, USA). Paired-end sequencing was performed using the Illumina NovaSeq6000 platform (*n* = 3). The global mRNA expression related to amino acids, fatty acids, and glucose transporters was visualized using a volcano plot and heat map.

*Isolation of mononuclear cells from the small intestine of mice*

To prevent blood contamination in the small intestine, systemic perfusion with heparinized saline was performed before harvesting or washing the tissue with PBS. Samples were stored in cold 2% FBS in RPMI until use in the experiments. The following experiments were performed on the day of euthanasia: Intestinal lamina propria (LPL) mononuclear cells were isolated using the Lamina Propria Dissociation Kit (130-097-410; Miltenyi Biotec, Germany), following the manufacturer’s instructions. Cell pellets were resuspended in 5 mL of 40% Percoll^®^ and the cell suspension was slowly added to the upper portion of centrifuge tubes, which contained a bottom layer of 5 mL of 80% Percoll^®^. Density gradient centrifugation (420×*g*, 20 min) was performed, and mononuclear cells in the middle layer were gently extracted with a 1 mL pipette. The extracted mononuclear cells were washed twice with 2% FBS/PBS.

*Tissue preparation and flow cytometry*

The cell suspension obtained, as described in the previous section, was preincubated with mouse BD™ Block purified anti-mouse CD16/CD32 mAb (394656; clone: 2.4G2; 1/100; BD Biosciences, Piscataway, NJ, USA) for 10 min at 22 °C. The antibodies used to gate innate lymphoid cells are mentioned below. Cell suspensions were incubated with a mixture of Biotin-CD3e (100304; clone: 145-2C11; 1/200; eBioscience, San Diego, CA, USA), Biotin-CD45R/B220 (103204; clone: RA3–6B2; 1/200; eBioscience), Biotin-Gr-1 (108404; clone: RB6-8C5; 1/200; eBioscience), Biotin-CD11c (117304; clone: N418; 1/200; eBioscience), Biotin-CD11b (101204; clone: M1/70; 1/200; eBioscience), Biotin-Ter119 (116204; clone: TER-119; 1/200; eBioscience), Biotin-FceRIa (134304; clone: MAR-1; 1/200; eBioscience), Brilliant Violet 510™ -Streptavidin (405233; 1/500; eBioscience), PE-Cy7-CD127 (135014; clone: A7R34; 1/100; eBioscience), Pacific Blue-CD45 (103116; clone: 30-F11; 1/100; eBioscience), and Fixable Viability Dye eFluor 780 (1/400; eBioscience) for 20 min at 4 °C. The cell suspension was washed twice with 2% FBS/PBS and fixed with a fixation buffer (420801; BioLegend, CA, USA) for 30 min. After washing with 2% FBS/PBS, the cell suspension was incubated with a mixture of PE-GATA-3 (clone TWAJ, 1/50; eBioscience), APC-RORγ (clone AFKJS-9, 1/50; eBioscience), and FITC-T-bet (clone 4B10, 1/50; BioLegend) (S8,9) (**Figure S1**). The following antibodies were used to gate M1 and M2 macrophages: FITC-CD206 (MA516870; clone: MR5D3, 1/50; eBioscience), PE-F4/80 (12480182; clone: BM8, 1/50; eBioscience), APC-CD45.2 (17045482; clone: 104, 1/50; eBioscience), PE-Cy7-CD11c (25011482; clone: N418, 1/50; eBioscience), and APC-Cy7-CD11b (47011282; clone: M1/70, 1/50; eBioscience) (S10) (**Figure S2**). The stained cells were analyzed using a Canto II flow cytometer, and the data were analyzed using the FlowJo software (version 10; TreeStar, Ashland, OR, USA) (*n* = 6).

*Measurement of amino acid and organic acid levels in the serum and skeletal muscle samples and short-chain fatty acid (SCFAs) levels in the serum and fecal samples*

The composition of amino acids and organic acids in murine sera and gastrocnemius muscle and of SCFAs in murine sera and feces was determined using gas chromatography-mass spectrometry (GC/MS) performed on an Agilent 7890B/7000D system (Agilent Technologies, Santa Clara, CA, USA). We homogenized 20 mg of gastrocnemius muscle and feces in 500 µL of acetonitrile and 500 µL of distilled water by grinding in a ball mill (4000 rpm for 2 min). The serum samples were not ball-milled before proceeding to the next step. The samples were then shaken at 1000 rpm for 30 min at 37 °C and centrifuged at 14,000 rpm for 3 min at room temperature. The supernatant (500 μL) was separated, and 500 μL of acetonitrile was added to it; the mixture was shaken at 1000 rpm for 3 min at 37 °C. After centrifugation at 14,000 rpm for 3 min at room temperature, the pH of the mixture was adjusted to 8 with 0.1 mol/L NaOH, after which amino acids, organic acids, and SCFAs were extracted. The concentrations of amino acids, organic acids, and SCFAs were determined using GC/MS employing the online solid-phase extraction (SPE) method. In the SPE-GC system SGI-M100 (AiSTI SCIENCE, Wakayama, Japan), SPE and injection into the GC/MS system were automatically performed after the sample was added to the vial and set on an autosampler tray. Flash-SPE ACXs (AiSTI SCIENCE) was used for solid phase stratification. To measure the levels of amino acids and organic acids, 50 µL aliquots of each of the aforementioned sample extracts were loaded onto the solid phase and washed with acetonitrile and water (1:1). The samples were then dehydrated with acetonitrile and impregnated with 4 μL of 0.5% methoxyamine–pyridine solution. Thereafter, N-methyl-N-trimethylsilyltrifluoroacetamide was supplied to the solid phase to perform methoxylation and trimethylsilylation during derivatization and eluted with hexane. The final product was injected through the PTV injector, LVI-S250 (AiSTI SCIENCE), and the temperature was maintained at 220 °C for 0.5 min, increased gradually at 50 °C/min to 290 °C, and then held there for 16 min. The samples were loaded onto a capillary column, Vf-5 ms (30 m × 0.25 mm [inner diameter] × 0.25 μm [membrane thickness]; Agilent Technologies). The column temperature was maintained at 80 °C for 3 min, then increased gradually at 25 °C/min to 190 °C, at 3 °C/min to 220 °C, and at 15 °C/min to 310 °C, and held there for 4.6 min. The sample was injected in the split mode at a split ratio of 50:1. To measure SCFAs, 50 μL aliquots of each of the aforementioned sample extracts were loaded onto the solid phase and washed with acetonitrile and water (1:1). The samples were then dehydrated with acetone, impregnated with 4 μL of N-tert-butyldimethylsilyl-N-methyltrifluoroacetamide–toluene solution (1:3), and eluted with hexane after derivatization on the solid phase. The final product was injected through the PTV injector, LVI-S250, and the temperature was maintained at 150 °C for 0.5 min, increased gradually at 25 °C/min to 290 °C, and then held there for 16 min. The samples were loaded onto a capillary column, Vf-5 ms (30 m × 0.25 mm [inner diameter] × 0.25 μm [membrane thickness]; Agilent Technologies). The column temperature was maintained at 60 °C for 3 min, increased gradually at 10 °C/min to 100 °C and at 20 °C/min to 310 °C, and then held there for 7 min. The sample was injected in the split mode at a split ratio of 20:1. Amino acids, organic acids, and SCFAs were detected in the scan mode (m/z, 70–470). All results were normalized to the peak heights of norleucine, adipic acid, and tetradeuteroacetic acid (0.01 mM) for amino acids, organic acids, and SCFAs, respectively (S11) (*n* = 6).

*16S rRNA sequencing*

A QIAamp DNA Feces Mini Kit (Qiagen, Venlo, The Netherlands) was used to extract microbial DNA from frozen appendicular fecal samples, according to the manufacturer’s instructions. The V3-V4 region of the 16S rRNA gene was amplified from the DNA using a bacterial universal primer set (341F and 806R). PCR was performed using EF-Taq (Korea, Solgent) with 20 ng of genomic DNA as a template in a 30 µL reaction mixture with the following thermocycling parameters: 95 °C for 2 min for activating Taq polymerase, followed by 35 cycles at 95 °C, 55 °C, and 72 °C for 1 min each, and a final 10 min step at 72 °C. Amplification products were purified using a multiscreen filter plate (Millipore Corp., Billerica, MA, USA). A MiSeq sequencer (Illumina, CA, USA) was used for 16S rRNA sequencing according to the manufacturer’s instructions (Macrogen, Seoul, Korea). For quality filtering of the sequences, we used QIIME version 1.9.1. (S12). Barcodes or primers with scores of less than 75% were excluded from the files. The number of operational taxonomic units (OTUs) was determined using the UCLUST algorithm at 97% similarity (S13). In addition, BLAST (UNITE, 2017) was used for the taxonomic assignment of 16S rRNAs with the UNITE sequence set of the Greengenes core-set-aligned with UCLUST and ITS.

The Kyoto Encyclopedia of Genes and Genomes (KEGG) ortholog abundance predictions were obtained using the Phylogenetic Investigation of Communities by Reconstruction of Unobserved States (PICRUSt2) software (S14).

The relative abundance of phyla in the groups was evaluated using one-way ANOVA with Holm–Šídák multiple-comparison test. Alpha diversity (defined as the diversity within an individual sample) was analyzed using the Chao1 (S15), Shannon (S16), and Gini–Simpson indices (S17).

The relative abundance of bacterial genera between the groups was evaluated using linear discriminant analysis (LDA) coupled with effect size measurements (LEfSe) (<http://huttenhower.sph.harvard.edu/lefse/>, accessed on May 15, 2022) (S18). With a normalized relative abundance matrix, LEfSe showed taxa with significantly different abundance, and the effect size of the feature was estimated using LDA. A *p*-value threshold of 0.05 (Wilcoxon rank-sum test) and an effect size threshold of 2 were used for all biomarkers discussed in this study.

In addition, principal coordinate analysis (PCoA) was performed to determine the effectiveness of FMT, and　nonhierarchical *K*-means cluster analysis was performed, with the number of clusters to be generated prespecified as 2,　using the Tinn-R Gui version 1.19.4.7, R version 1.36 (S19).

**References**

S1. Ochoa-Repáraz J, Mielcarz DW, Ditrio LE, Burroughs AR, Foureau DM, Haque-Begum S, et al. Role of gut commensal microflora in the development of experimental autoimmune encephalomyelitis. J Immunol [Internet]. 2009 Nov 15 [cited 2022 May 23];183(10):6041–50. Available from: https://pubmed.ncbi.nlm.nih.gov/19841183/

S2. Wang S, Huang M, You X, Zhao J, Chen L, Wang L, et al. Gut microbiota mediates the anti-obesity effect of calorie restriction in mice. Sci Rep [Internet]. 2018 Dec 1 [cited 2022 May 23];8(1). Available from: https://pubmed.ncbi.nlm.nih.gov/30158649/

S3. Okamura T, Hamaguchi M, Mori J, Yamaguchi M, Mizushima K, Abe A, et al. Partially Hydrolyzed Guar Gum Suppresses the Development of Sarcopenic Obesity. Nutrients [Internet]. 2022 Mar 1 [cited 2022 Oct 20];14(6). Available from: https://pubmed.ncbi.nlm.nih.gov/35334814/

S4. Kotani K, Maekawa M KT. [Reestimation of aspartate aminotransferase (AST)/alanine aminotransferase (ALT) ratio based on JSCC consensus method--changes of criteria for a differential diagnosis of hepatic disorders following the alteration from Karmen method to JSCC method] - PubM. Nihon Shokakibyo Gakkai Zasshi [Internet]. 1994 [cited 2022 Feb 28];91:154–61. Available from: https://pubmed.ncbi.nlm.nih.gov/8114321/

S5. McGowan MW, Artiss JD, Strandbergh DR ZB. A peroxidase-coupled method for the colorimetric determination of serum triglycerides - PubMed. Clin Chem [Internet]. 1983 [cited 2022 Feb 28];29:538–42. Available from: https://pubmed.ncbi.nlm.nih.gov/6825269/

S6. Christmass MA, Mitoulas LR, Hartmann PE, Arthur PG. A semiautomated enzymatic method for determination of nonesterified fatty acid concentration in milk and plasma. Lipids [Internet]. 1998 [cited 2022 Feb 28];33(10):1043–9. Available from: https://pubmed.ncbi.nlm.nih.gov/9832086/

S7. Motta JP, Flannigan KL, Agbor TA, Beatty JK, Blackler RW, Workentine ML, et al. Hydrogen sulfide protects from colitis and restores intestinal microbiota biofilm and mucus production. Inflamm Bowel Dis [Internet]. 2015 Mar 3 [cited 2021 Dec 28];21(5):1006–17. Available from: https://pubmed.ncbi.nlm.nih.gov/25738373/

S8. Molofsky AB, Nussbaum JC, Liang HE, van Dyken SJ, Cheng LE, Mohapatra A, et al. Innate lymphoid type 2 cells sustain visceral adipose tissue eosinophils and alternatively activated macrophages. J Exp Med [Internet]. 2013 Mar 11 [cited 2019 Mar 15];210(3):535–49. Available from: http://www.ncbi.nlm.nih.gov/pubmed/23420878

S9. Wang S, Li J, Wu S, Cheng L, Shen Y, Ma W, et al. Type 3 innate lymphoid cell: a new player in liver fibrosis progression. Clin Sci [Internet]. 2018 Dec 21 [cited 2019 Jul 13];132(24):2565–82. Available from: http://www.ncbi.nlm.nih.gov/pubmed/30459204

S10. Ono Y, Nagai M, Yoshino O, Koga K, Nawaz A, Hatta H, et al. CD11c+ M1-like macrophages (MΦs) but not CD206+ M2-like MΦ are involved in folliculogenesis in mice ovary. Sci Rep [Internet]. 2018 [cited 2019 Dec 29];8(1):8171. Available from: http://www.ncbi.nlm.nih.gov/pubmed/29802255

S11. Nakajima H, Nakanishi N, Miyoshi T, Okamura T, Hashimoto Y, Senmaru T, et al. Inulin reduces visceral adipose tissue mass and improves glucose tolerance through altering gut metabolites. Nutr Metab (Lond) [Internet]. 2022 Dec 1 [cited 2022 Oct 16];19(1). Available from: https://pubmed.ncbi.nlm.nih.gov/35902903/

S12. Caporaso JG, Kuczynski J, Stombaugh J, Bittinger K, Bushman FD, Costello EK, et al. QIIME allows analysis of high-throughput community sequencing data. Vol. 7, Nature Methods. 2010. p. 335–6.

S13. Edgar RC. Search and clustering orders of magnitude faster than BLAST. Bioinformatics. 2010 Aug 12;26(19):2460–1.

S14. Douglas GM, Maffei VJ, Zaneveld JR, Yurgel SN, Brown JR, Taylor CM, et al. PICRUSt2 for prediction of metagenome functions. Nat Biotechnol [Internet]. 2020 Jun 1 [cited 2022 Oct 18];38(6):685–8. Available from: https://pubmed.ncbi.nlm.nih.gov/32483366/

S15. Chao A, Chazdon RL, Colwell RK, Shen TJ. Abundance-based similarity indices and their estimation when there are unseen species in samples. Biometrics [Internet]. 2006 Jun [cited 2022 May 16];62(2):361–71. Available from: https://pubmed.ncbi.nlm.nih.gov/16918900/

S16. Shannon C.E. WW. The mathematical theory of communication. University of Illinois Press. 1949;pp 1–117.

S17. Simpson EH. Measurement of Diversity. Nature 1949 163:4148 [Internet]. 1949 [cited 2022 May 16];163(4148):688–688. Available from: https://www.nature.com/articles/163688a0

S18. Segata N, Izard J, Waldron L, Gevers D, Miropolsky L, Garrett WS, et al. Metagenomic biomarker discovery and explanation. Genome Biol [Internet]. 2011 Jun 24 [cited 2021 Aug 18];12(6). Available from: https://pubmed.ncbi.nlm.nih.gov/21702898/

S19. Ding C, He X. K-means clustering via principal component analysis. Proceedings, Twenty-First International Conference on Machine Learning, ICML 2004. 2004;225–32.
