# Supplementary material for: Milk protects against sarcopenic obesity due to increase in the genus Akkermansia in faeces of db/db mice
Source: J Cachexia Sarcopenia Muscle. 2023 May 2;14(3):1395–409. doi: 10.1002/jcsm.13245 (PMC10235896; doi:10.1002/jcsm.13245)
Supplement: Supplementary file 2 — Table S1. Primer sequences Table S2. KEGG pathway in four groups Table S3. KEGG pathway Class II in four groups [file JCSM-14-1395-s003.docx]

**Supplementary Table 1. Primer sequences**

| Gene symbol | Accession number | Primer sequence (5' to 3') | Primer sequence (3' to 5') |
| --- | --- | --- | --- |
| *Foxo1* | Mm00490671_m1 | CTGCAGATCCCGTAAGACG | GGTCACCGTGTCTAAGGAG |
| *Mstn* | Mm01254559_m1 | ACTGCACCTCTCGATAGAAC | ACTTAGTGCTGTGTGTGTG |
| *Fbxo32* | Mm00499523_m1 | ACGACGTCGCAGCCAAGAAG | TCCATGGCGCTCCTTCGTAC |
| *Trim63* | Mm01185221_m1 | CCTTCCTCTCTCAAGTGGCC | TGCGCCCTCAAGGCCTCTGC |
| *Gapdh* | Mm99999915_g1 | CATGGCCTTCCGTGTTCCTA | TGTCATCATACTTGGCAGGTT |

**Supplementary Table 2. KEGG pathway in four groups**

| KEGG pathway Class I | Milk- | Milk+ | *p* value | FMT(Db) | FMT(M) | *p* value |
| --- | --- | --- | --- | --- | --- | --- |
| Cellular Processes | 3.8(0.2) | 4.6(0.2) | 0.008 | 3.8(0.3) | 4(0.5) | 0.658 |
| Environmental Information Processing | 13(0.1) | 14.3(0.3) | 0.001 | 11.3(0.7) | 12.9(0.3) | 0.022 |
| Genetic Information Processing | 19.5(0.1) | 19.3(0.2) | 0.159 | 20(0.4) | 19.9(0.3) | 0.841 |
| Human Diseases | 0.8(0.0) | 0.8(0.0) | 0.969 | 0.8(0.0) | 0.8(0.0) | 0.017 |
| Metabolism | 48.1(0.2) | 46.2(0.3) | 0.001 | 49.1(0.9) | 47.4(0.5) | 0.040 |
| Organismal Systems | 0.8(0.0) | 0.7(0.0) | 0.045 | 0.8(0.0) | 0.8(0.0) | 0.006 |
| Unclassified | 14.1(0.1) | 14(0.1) | 0.472 | 14.1(0.2) | 14.2(0.1) | 0.483 |

Data are expressed as mean (SD). Student's paired t-test were conducted between two groups.

**Supplementary Table 3. KEGG pathway Class II in four groups**

| KEGG Pathway – Class II | Milk- | Milk+ | *p* value | FMT(Db) | FMT(M) | *p* value |
| --- | --- | --- | --- | --- | --- | --- |
| Amino Acid Metabolism | 9.63(0.02) | 9.20(0.10) | 0.002 | 9.81(0.22) | 9.56(0.16) | 0.191 |
| Biosynthesis of Other Secondary Metabolites | 1.06(0.04) | 0.89(0.05) | 0.009 | 1.06(0.07) | 0.95(0.04) | 0.060 |
| Cancers | 0.12(0.00) | 0.12(0.00) | 0.572 | 0.14(0.01) | 0.13(0.00) | 0.032 |
| Carbohydrate Metabolism | 10.41(0.16) | 10.00 (0.17) | 0.035 | 10.25(0.23) | 10.16(0.24) | 0.679 |
| Cardiovascular Diseases | 0.00 (0.00) | 0.00 (0.00) | 0.374 | 0.00 (0.00) | 0.00 (0.00) | 0.120 |
| Cell Communication | 0.00 (0.00) | 0.00 (0.00) | - | 0.00 (0.00) | 0.00 (0.00) | - |
| Cell Growth and Death | 0.53(0.01) | 0.55(0.01) | 0.084 | 0.59(0.02) | 0.56(0.01) | 0.101 |
| Cell Motility | 2.77(0.22) | 3.70(0.21) | 0.006 | 2.74(0.29) | 3.01(0.50) | 0.477 |
| Cellular Processes and Signaling | 3.95(0.05) | 3.84(0.01) | 0.027 | 3.93(0.10) | 3.98(0.06) | 0.475 |
| Circulatory System | 0.00 (0.00) | 0.00 (0.00) | 0.374 | 0.00 (0.00) | 0.00 (0.00) | 0.120 |
| Digestive System | 0.04(0.00) | 0.03(0.00) | 0.013 | 0.04(0.00) | 0.04(0.00) | 0.027 |
| Endocrine System | 0.33(0.01) | 0.32(0.01) | 0.218 | 0.37(0.01) | 0.33(0.00) | 0.002 |
| Energy Metabolism | 5.83(0.10) | 5.87(0.04) | 0.564 | 6.24(0.10) | 6.05(0.05) | 0.046 |
| Environmental Adaptation | 0.16(0.01) | 0.18(0.00) | 0.019 | 0.17(0.01) | 0.17(0.00) | 0.248 |
| Enzyme Families | 2.19(0.02) | 2.08(0.03) | 0.004 | 2.08(0.04) | 2.07(0.05) | 0.892 |
| Excretory System | 0.03(0.00) | 0.02(0.00) | 0.003 | 0.03(0.00) | 0.02(0.00) | 0.028 |
| Folding, Sorting and Degradation | 2.49(0.00) | 2.45(0.04) | 0.127 | 2.71(0.09) | 2.55(0.03) | 0.044 |
| Genetic Information Processing | 2.43(0.03) | 2.55(0.04) | 0.013 | 2.56(0.06) | 2.59(0.05) | 0.551 |
| Glycan Biosynthesis and Metabolism | 3.18(0.11) | 2.72(0.02) | 0.002 | 3.25(0.15) | 2.81(0.05) | 0.010 |
| Immune System | 0.10(0.00) | 0.09(0.00) | 0.043 | 0.10(0.00) | 0.10(0.00) | 0.083 |
| Immune System Diseases | 0.04(0.00) | 0.03(0.00) | 0.012 | 0.04(0.00) | 0.03(0.00) | 0.042 |
| Infectious Diseases | 0.36(0.00) | 0.38(0.01) | 0.054 | 0.39(0.00) | 0.38(0.02) | 0.360 |
| Lipid Metabolism | 2.82(0.05) | 2.73(0.03) | 0.054 | 2.93(0.07) | 2.82(0.04) | 0.083 |
| Membrane Transport | 10.99(0.09) | 12.12(0.21) | 0.001 | 9.36(0.59) | 10.88(0.20) | 0.013 |
| Metabolic Diseases | 0.12(0.00) | 0.10(0.00) | 0.005 | 0.13(0.00) | 0.11(0.00) | 0.038 |
| Metabolism | 2.75(0.06) | 2.68(0.02) | 0.124 | 2.69(0.05) | 2.68(0.03) | 0.755 |
| Metabolism of Cofactors and Vitamins | 4.3(0.05) | 4.29(0.04) | 0.858 | 4.56(0.13) | 4.34(0.10) | 0.068 |
| Metabolism of Other Amino Acids | 1.54(0.03) | 1.39(0.03) | 0.003 | 1.56(0.06) | 1.46(0.05) | 0.085 |
| Metabolism of Terpenoids and Polyketides | 1.58(0.01) | 1.58(0.02) | 0.989 | 1.78(0.09) | 1.68(0.03) | 0.136 |
| Nervous System | 0.11(0.00) | 0.09(0.00) | 0.012 | 0.10(0.00) | 0.10(0.01) | 0.471 |
| Neurodegenerative Diseases | 0.15(0.00) | 0.15(0.01) | 0.944 | 0.14(0.00) | 0.13(0.01) | 0.170 |
| Nucleotide Metabolism | 4.03(0.03) | 3.93(0.02) | 0.012 | 4.10(0.07) | 4.02(0.04) | 0.187 |
| Poorly Characterized | 4.95(0.03) | 4.94(0.03) | 0.739 | 4.90(0.11) | 4.93(0.02) | 0.680 |
| Replication and Repair | 8.79(0.04) | 8.61(0.09) | 0.033 | 9.09(0.22) | 8.98(0.17) | 0.535 |
| Sensory System | 0.00 (0.00) | 0.00 (0.00) | - | 0.00 (0.00) | 0.00 (0.00) | - |
| Signal Transduction | 1.79(0.04) | 2.04(0.10) | 0.018 | 1.74(0.12) | 1.84(0.14) | 0.425 |
| Signaling Molecules and Interaction | 0.20(0.01) | 0.17(0.00) | 0.004 | 0.19(0.01) | 0.18(0.01) | 0.340 |
| Transcription | 2.62(0.05) | 2.66(0.05) | 0.463 | 2.34(0.10) | 2.62(0.10) | 0.026 |
| Translation | 5.59(0.05) | 5.60(0.06) | 0.858 | 5.88(0.22) | 5.79(0.05) | 0.554 |
| Transport and Catabolism | 0.52(0.04) | 0.37(0.02) | 0.003 | 0.49(0.05) | 0.41(0.04) | 0.095 |
| Xenobiotics Biodegradation and Metabolism | 1.51(0.01) | 1.53(0.01) | 0.028 | 1.52(0.03) | 1.53(0.03) | 0.931 |

Data are expressed as mean (SD). Student's paired t-test were conducted between two groups.
