# Supplementary material for: Milk protects against sarcopenic obesity due to increase in the genus Akkermansia in faeces of db/db mice
Source: J Cachexia Sarcopenia Muscle. 2023 May 2;14(3):1395–409. doi: 10.1002/jcsm.13245 (PMC10235896; doi:10.1002/jcsm.13245)
Supplement: Supplementary file 3 — Figure S1. Strategy for innate lymphoid cells (ILCs). Representative flow cytometry plots of liver CD45+ Live & Dead‐ Lin‐ CD127+ RORg‐ GATA‐3‐ T‐bet+ ILC1s, CD45+ Live & Dead‐ Lin‐ CD127+ RORg‐ GATA‐3+ ILC2s and CD45+ Live & Dead‐ Lin‐ CD127+ RORg+GATA‐3‐ ILC3s in each group at 16‐weeks of age. Figure S2. Strategy for macrophages. Representative flow cytometry plots of liver CD45+ F4/80+ CD206‐ CD11c+ M1 macrophages and CD45+ F4/80+ CD206+ CD11c‐ M2 macrophages in each group at 16 weeks of age. [file JCSM-14-1395-s002.pdf]

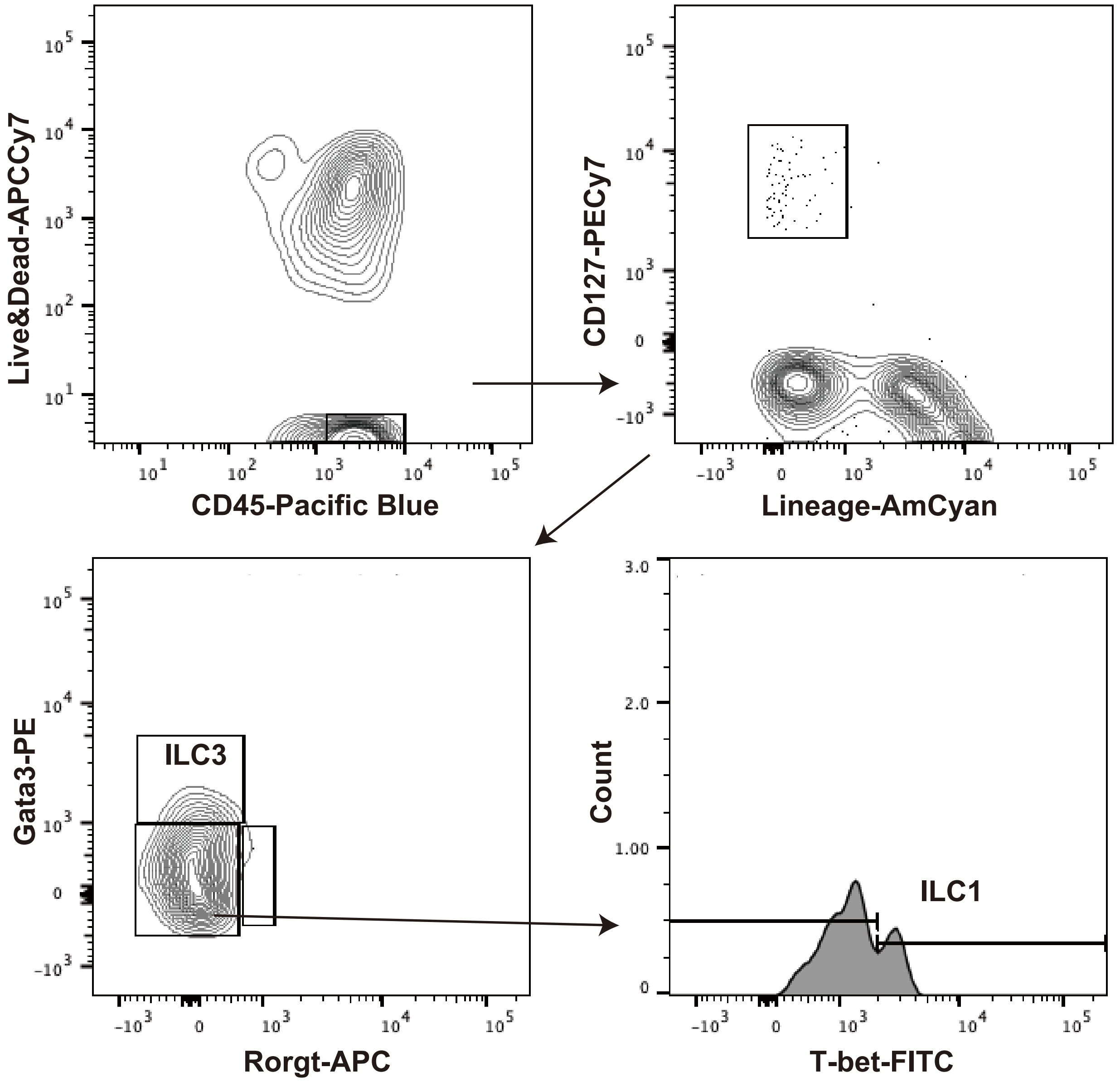

Supplementary Figure 1. Strategy for innate lymphoid cells (ILCs)

Representative flow cytometry plots of liver CD45+ Live & Dead- Lin- CD127+ RORg- GATA-3- T-bet+ ILC1s, CD45+ Live & Dead- Lin- CD127+ RORg- GATA-3+ ILC2s and CD45+ Live & Dead- Lin- CD127+ RORg+ GATA-3- ILC3s in each group at 16-weeks of age.

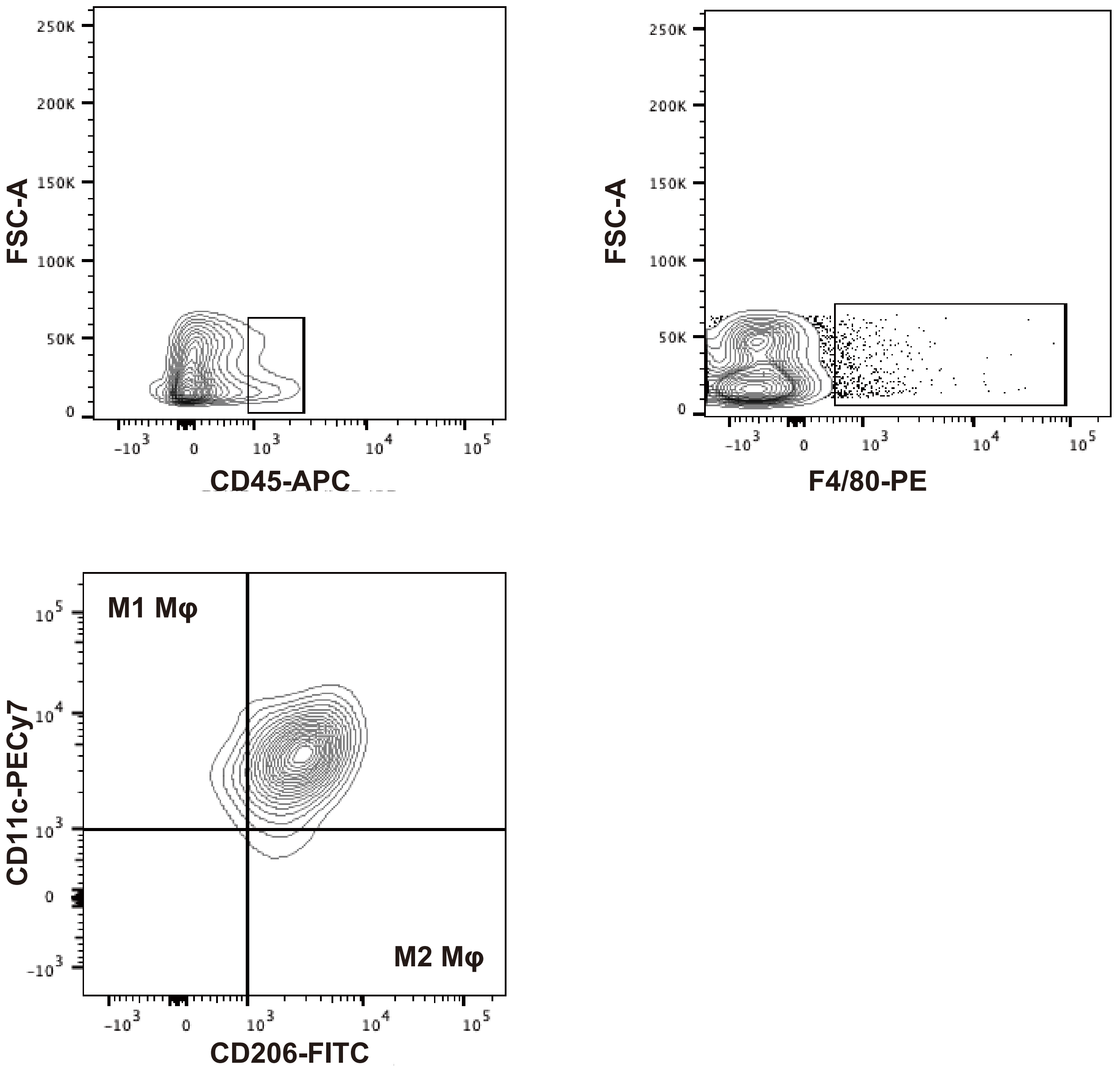

Supplementary Figure 2. Strategy for macrophages

Representative flow cytometry plots of liver CD45+ F4/80+ CD206- CD11c+ M1 macrophages and CD45+ F4/80+ CD206+ CD11c- M2 macrophages in each group at 16 weeks of age.
